# Prevalence and sociodemographic factors associated with hyperemesis gravidarum in pregnant women in European Arctic Russia, 2006–2018

**DOI:** 10.1186/s12884-026-08699-w

**Published:** 2026-01-31

**Authors:** Natalia A. Treskina, Vitaly A. Postoev, Anna A. Usynina, Andrej M. Grjibovski, Elisabeth Darj, Jon Øyvind Odland

**Affiliations:** 1 Department of Public Health and Nursing, Norwegian University of Science and Technology, 8900, Trondheim, NO-7491 Norway; 2 Almazov National Medical Research Centre, Saint Petersburg, Russia; 3 Department of Research Methodology, Northern State Medical University, Arkhangelsk, Russia; 4 Department of Neonatology and Perinatology, Northern State Medical University, Arkhangelsk, Russia; 5 Reaviz Universtiy, Saint Petersburg, Russia; 6 Department of Epidemiology and Modern Vaccination Technologies, I.M. Sechenov First Moscow State Medical University, Moscow, Russia; 7 Department of Health Policy and Management, Al Farabi Kazakh National University, Almaty, Kazakhstan; 8 Department of Healthcare Organization and Preventive Medicine, North-Eastern Federal University, Yakutsk, Russia; 9 Department of Women’s and Children’s Health, Uppsala University, Uppsala, Sweden; 10 Department of General Hygiene, I.M. Sechenov First Moscow State Medical University, Moscow, Russia; 11 Institute of Ecology, National Research University Higher School of Economics, Moscow, Russia

**Keywords:** Birth registry, Body mass index, Hyperemesis gravidarum, Russia, Sociodemographic factors

## Abstract

**Background:**

Hyperemesis gravidarum is defined as intractable nausea and vomiting which occurs in 0.3–3% of all pregnancies. Data on hyperemesis in Russia are scarce. This study estimates the prevalence of hyperemesis gravidarum in pregnant women in two Arctic regions of European Russia.

**Methods:**

A retrospective registry-based study was used. Data were collected from two population-based birth registries between 2006 and 2018. Bivariate associations between the prevalence of hyperemesis and potential determinants were studied via chi-squared tests. To assess factors contributing to the changes in the prevalence of hyperemesis over time, logistic regression models were applied. In total, 124,538 births composed the study base.

**Results:**

The overall prevalence of hyperemesis was 2.4%. An inverse association was observed between maternal age and the prevalence of hyperemesis. Teenagers and women aged 20–24 years were more likely to have hyperemesis than women aged 25–29 years were (aOR = 1.32; 95% CI: 1.13 − 1.55 and aOR = 1.20; 95% CI: 1.08 − 1.34, respectively). The level of education did not influence the likelihood of developing hyperemesis. Being married, being primiparous, being underweight and having a female child were associated with hyperemesis. Women who reported no smoking during pregnancy were more likely to have hyperemesis after adjustment for other studied factors.

**Conclusions:**

The prevalence of hyperemesis in European Arctic Russia is greater than that in neighboring Nordic countries and varies over time. Changes in the prevalence of hyperemesis over time cannot be explained by changes in maternal sociodemographic characteristics during the study period.

## Background

Nausea and vomiting during pregnancy may affect the health of a pregnant woman from approximately 6–8 weeks of gestation, subsiding by weeks 16–20, which occurs in 70–80% of cases [1, 2]. The quality of life of pregnant women may be affected, and health care costs and time spent out of work can increase. Hyperemesis gravidarum (HG) is defined as intractable nausea and vomiting starting before the end of the 22nd week of gestation (according to the International Classification of Disease-10 (ICD-10)) and occurs in 0.3–3% of all pregnancies [1, 3]. In 2021 an international Windsor definition of HG was published according to that HG starts before 16 weeks of gestation [4]. HG malnutrition may lead to dehydration, ketoacidosis and weight loss [2, 5]. Cardiac, renal or neuromuscular complications rarely develop [6, 7]. A British study of more than 8 million pregnancies revealed that HG was also associated with anemia, preeclampsia, eclampsia, venous thromboembolism, preterm birth and babies small for gestational age [8]. 

The etiology of HG is not clearly understood. Currently, increased levels of growth-differentiation factor 15 (GDF15) is considered to be a risk factor for HG, especially, hypersensitivity to the rise of GDF15 during pregnancy [9, 10]. It was revealed that the large amount of GDF15 in maternal blood is occurred from the feto-placental unit [10]. The severity of HG is influenced by the maternal sensitivity to GDF15 and is determined by prepregnancy exposure to the hormone and level of fetal derived GDF15, thus nonpregnant woman with low levels of GDF15 may have increased risk of HG developing and vice versa [10]. A number of sociodemographic, behavior factors and health related conditions have been found to be associated with HG, including young age [11, 12], unmarried status [13], primiparity [11, 14], multiple gestation [11, 12, 15], female fetus [12, 15, 16], and under- or overweight [15, 17]. Smoking before and during pregnancy is not considered a risk factor for HG and may even reduce its risk [11, 15, 17, 18]. 

The prevalence of HG varies across countries. In a Swedish study, 0.3% [19] of women were diagnosed with HG, 0.5% [20] in California, 0.8% [22] in Canada, 0.9% [12] in Norway, 1.3% [15] in Finland, 2.1% [21] in the United Kingdom, and 3.9% [22] in Malaysia. Different designs of the presented studies and absence of international consensus on diagnostic criteria for HG before 2021 [4], could partly influence on this variance.

Time trends of HG were presented in a number of studies. In Norway, the occurrence of HG occurrence was higher in 1977–1986 and 1997–2005 than in the year of delivery in 1967–1976 and 1987–1996 [12]. In England, the number of hospital admissions and antiemetic prescriptions for women with HG increased continuously from 1998 to 2013 [21]. In the United States, the proportion of pregnant women with HG increased from 4.2% to 5.7% between 2006 and 2014 [23]. In Finland, the annual incidence of HG in 2005–2017 varied between 1.2% and 1.5% [15]. The cause of the variation in HG over time is not known.

Data on HG in Russia are scarce and, to our knowledge, no one has published with a sufficient sample size in English or Russian languages. The true incidence of HG is difficult to estimate because of a lack of official statistics, various diagnostic approaches and different maternal attitudes toward medical care [24]. 

The lack of data on HGs in Russia warrants a large study with a long observation period. Medical birth registries in Northwest Russia contain data on maternal, pregnancy and fetal characteristics [25, 26], providing a unique opportunity to investigate the prevalence of HG and associated factors in this part of the world.

The aim of this study was to assess the prevalence of HG and its changes in pregnant women in 2006–2018 using data from population-based birth registries in two Arctic regions of European Russia. Moreover, we hypothesized that the changes in the prevalence of HG may be explained by changes in maternal sociodemographic characteristics over time.

## Methods

### Study design and sample size

This was a registry-based retrospective cohort study. Data on all births in the Murmansk and Arkhangelsk Counties of Russia after 22 weeks of gestation were collected from the Murmansk County Birth Registry (MCBR) and the Arkhangelsk County Birth Registry (ACBR). The prospective registration of pregnancy outcomes in the MCBR began on the 1 st of January 2006 [25]. The ACBR contains prospective data from the 1 st of January, 2012 [26]. The registry form of the medical birth registry of Norway was used as a basis for the birth registration forms in Russia, containing information on maternal sociodemographic, lifestyle and behavior characteristics; maternal health before and during pregnancy; and information about the delivery and conditions of newborns. A depersonalized electronic database was constructed from the paper registration forms. Data from the database were used for research purposes. Information on the implementation and validity of the registries for epidemiological studies has been published previously [25, 26]. 

**Fig. 1:**
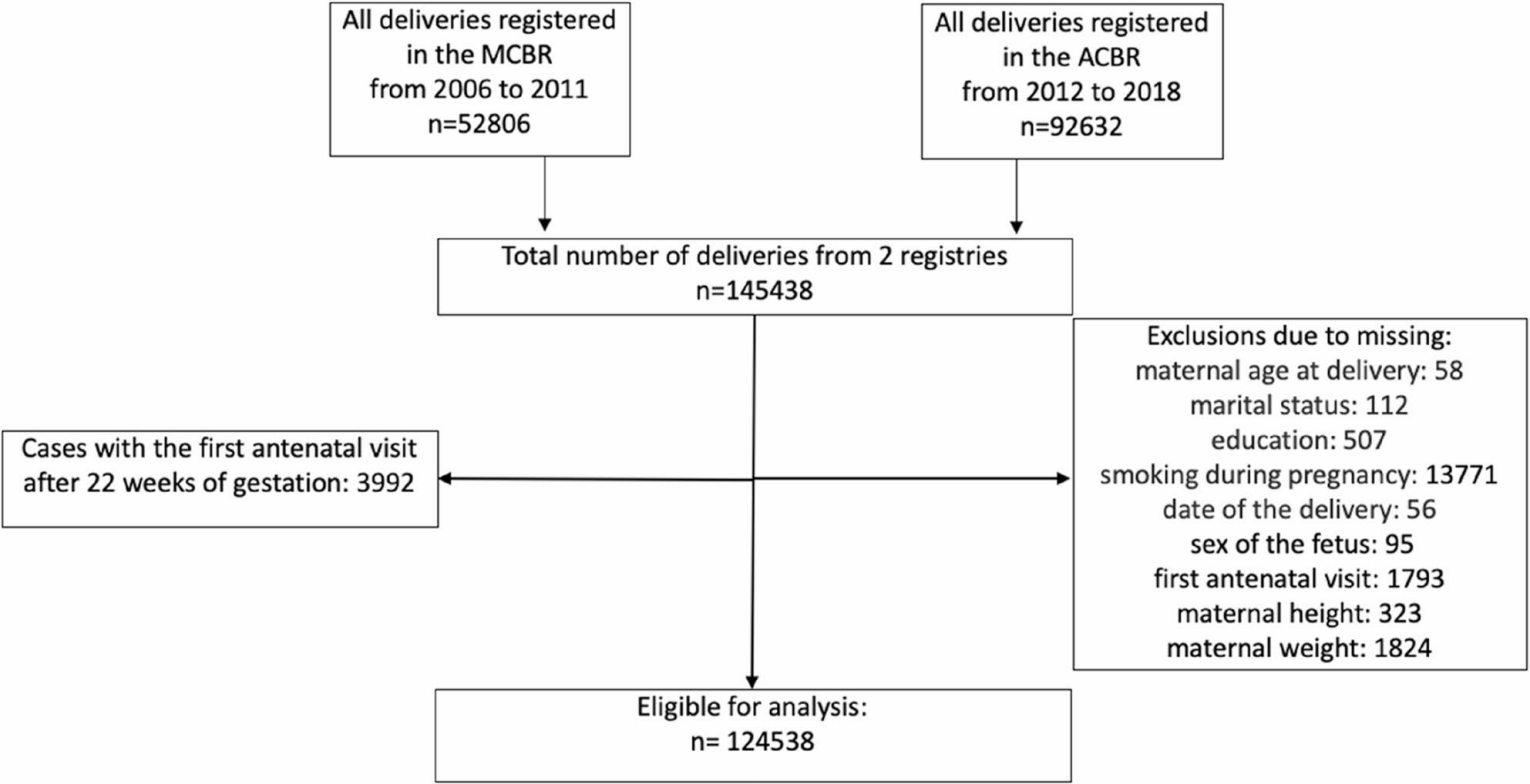
Flow chart of the sampling procedure

Data concerning all deliveries (*n* = 145438) during the period 2006–2018 from the MCBR or the ACBR were obtained. Deliveries with missing data on maternal age at delivery, year of birth, marital status, education, smoking status during pregnancy, sex of the child, data on the first antenatal visit, and maternal height and weight at the first antenatal visit were excluded from the study. Patients whose first antenatal visit occurred after 22 weeks of gestation were also excluded. The study was conducted on 124,538 deliveries and is presented in Fig. 1.

### Variables and statistical analysis

Data from the two birth registries were merged into one database according the same fields: maternal date of birth; child’s birth date; parity; plurality; civil status; education; occupation; maternal tobacco smoking during pregnancy; sex of the child; data on the first antenatal visit; maternal height and weight at the first antenatal visit; and data on HG in ICD-10 codes from the files of the birth registries (ICD-10 codes O21.0, O21.1 and O 21.9).

The maternal age at delivery was categorized into five groups: < 20 years, 20–24 years, 25–29 years, 30–34 years, and ≥ 35 years. The time periods were divided into 4 periods: 2006–08, 2009–11, 2012–14 and 2015–18. The education of the mother was classified into 5 categories: primary (classes 1–9) or none, secondary (classes 10–11), vocational, higher education and unknown. Maternal occupation was divided into 2 groups: employed or unemployed, the latter including students. Maternal parity was as follows: nullipara or multipara. By plurality, pregnancies were divided into single pregnancies and multiple pregnancies. Marital status was categorized into three groups: married (officially registered marriage), cohabiting, and unmarried (including single, divorced, widows). The smoking habits were registered according to self-reported information. Maternal smoking during pregnancy was categorized as “yes” or “no”. Sex of the child: male and female. The BMI at the first antenatal visit was calculated by dividing weight (kg) by height squared (m^2^). The maternal prepregnancy BMI was categorized into six groups: underweight (BMI < 18.5 kg/m^2^), normal weight (BMI = 18.5–24.9 kg/m^2^), overweight (BMI = 25.0–29.9 kg/m^2^), obesity class I (BMI = 30.0–34.9 kg/m^2^), obesity class II (BMI = 35.0–39.9 kg/m^2^), and obesity class III (BMI > 40 kg/m^2^) [27].

The overall registered prevalence of HG was calculated for the studied period. Bivariate associations between the prevalence of HG across the four time periods and potential determinants were performed via Pearson’s chi-squared tests. To assess factors contributing to the changes in the prevalence of HG over time, logistic regression models were used. Time periods and maternal sociodemographic characteristics (maternal age, parity, plurality, civil status, maternal education and occupation, sex of the child, maternal smoking status and BMI) were included as independent categorical variables, and HG was included as a dichotomous dependent variable. For the odds ratios, 95% confidence intervals (CIs) were calculated. The first time period (2006–08), maternal age 25–29 years, education below 9 years, occupational status, primiparity, marital status, singleton pregnancy, nonsmoking status during pregnancy, male sex of the child, and normal weight were used as reference categories. Statistical analysis was conducted via IBM SPSS Statistics for Windows, version 28.0.1.0 (IBM Corp., Armonk, NY, USA) [28]. 

## Results

Overall, 2955 cases of HG from in- and outpatient departments were registered in the MCBR and the ACBR from 2006 to 2018, resulting in the registered prevalence of 2.4%. The highest prevalence of HG was 3.2% from 2012 to 2014.

The distributions of pregnant women included in the study according to the sociodemographic characteristics are presented in Table 1.

**Table 1:** Prevalence of hyperemesis gravidarum (HG) according to maternal sociodemographic characteristics from 2006–2018, *N* = 124,538

	*N*	%	*n with HG*	Prevalence (%)	*p* value^a^
Time periods					
2006-08	23310	18.7	635	2.7	
2009-11	24,758	19.9	465	1.9	< 0.001
2012-14	38,073	30.6	1216	3.2	
2015-18	38,397	30.8	639	1.7	
Age of the women (years)					
< 20	10,237	8.2	267	2.6	
20–24	25,263	20.3	697	2.8	
25–29	42,641	34.2	1000	2.3	< 0.001
30–34	31,095	25.0	688	2.2	
≥ 35	15,302	12.3	303	2.0	
Education of the women					
primary (class 1–9) or none	6047	4.9	110	1.8	
secondary (class 10–11)	23,835	19.1	532	2.2	< 0.001
vocational	49,992	40.1	1213	2.4	
higher education	44,343	35.6	1099	2.5	
unknown	321	0.3	1	0.3	
Occupation					
employed	92,846	74.6	2262	2.4	0.012
unemployed (incl.students)	31,692	25.4	693	2.2	
Parity					
nullipara	40,654	32.6	1056	2.6	< 0.001
multipara	83,884	67.4	1899	2.3	
Single/multiple pregnancy					
single pregnancy	123,175	98.9	2923	2.4	0.951
multiple pregnancy	1363	1.1	32	2.3	
Marital status					
married	93,021	74.7	2325	2.5	< 0.001
cohabiting	19,526	15.7	419	2.1	
unmarried	11,991	9.6	211	1.8	
Maternal smoking during pregnancy					
no	105,145	84.4	2641	2.5	< 0.001
yes	19,393	15.6	314	1.6	
Sex of the child					
male	64,288	51.6	1451	2.3	0.006
female	60,250	48.4	1504	2.5	
Prepregnancy BMI					
underweight	8365	6.7	253	3.0	< 0.001
normal weight	80,274	64.5	1933	2.4	
overweight	24,938	20.0	566	2.3	
obesity class I	8113	6.5	156	1.9	
obesity class II	2200	1.8	40	1.8	
obesity class III	648	0.5	7	1.1	
Total	124,538	100.0			

^a^Calculated via chi-squared tests

The highest prevalence of HG was in women in the < 20- and 20–24-year groups (2.6% and 2.8%, respectively) (Table 1). Women with vocational or higher education, who are married, who are primiparous, who are pregnant with a female fetus and who are underweight in terms of body composition demonstrated a greater prevalence of HG. Compared with smoking women, a higher prevalence of HG was revealed in nonsmoking women. Plurality was not significantly associated with HG.

After adjustment for potential confounders, teenagers and women aged 20–24 years were more likely to have HG than women aged 25–29 years were. A further increase in maternal age was associated with a decrease in the prevalence of HG, although it did not reach statistical significance (Table 2). In the crude analysis, women with vocational and higher education levels had a greater risk of developing HG, but after adjustment, this association was not statistically significant. Working status and primiparity were associated with a higher risk of HG even after adjustment. Unmarried and cohabiting women had a lower risk for HG development than married women did in the multivariable analysis. Female sex of the child and nonsmoking status during pregnancy were associated with a greater likelihood of experiencing HG after adjustment for all other studied factors.

**Table 2:** Crude and adjusted odds ratios for the prevalence of hyperemesis gravidarum according to sociodemographic characteristics over time

	Crude OR (95% CI)	Adjusted OR^1^ (95% CI)
Time periods		
2006-08	1.00	1.00
2009-11	0.68(0.60–0.77)	0.68(0.60–0.77)
2012-14	1.17(1.06–1.29)	1.17(1.06–1.29)
2015-18	0.60(0.54–0.67)	0.59(0.53–0.66)
Age of women (years)		
< 20	1.11(0.97–1.27)	1.32(1.13–1.55)
20–24	1.18(1.07–1.30)	1.20(1.08–1.34)
25–29	1.00	1.00
30–34	0.94(0.85–1.03)	0.96(0.87–1.06)
≥ 35	0.84(0.73–0.95)	0.89(0.78–1.02)
Education of the women		
primary (class 1–9) or none	1.00	1.00
secondary (class 10–11)	1.23(1.00–1.51.00.51)	1.12(0.90–1.39)
vocational	1.34(1.10–1.63)	1.19(0.97–1.47)
higher education	1.37(1.12–1.67)	1.16(0.93–1.44)
unknown	0.16(0.02–1.21)	0.15(0.02–1.09)
Occupation		
employed	1.00	1.00
unemployed (incl. students)	0.89(0.82–0.97)	0.90(0.82–0.99)
Parity		
nullipara	1.00	1.00
multipara	0.86(0.80–0.93)	0.91(0.84–0.98)
Single/multiple pregnancy		
single pregnancy	1.00	1.00
multiple pregnancy	0.98(0.69–1.40)	0.99(0.69–1.41)
Marital status		
married	1.00	1.00
cohabiting	0.85(0.77–0.95)	0.87(0.78–0.97)
unmarried	0.69(0.60–0.80)	0.71(0.61–0.82)
Maternal smoking during pregnancy		
no	1.00	1.00
yes	0.63(0.56–0.71)	0.68(0.60–0.77)
Sex of the child		
male	1.00	1.00
female	1.10(1.03–1.19)	1.11(1.03–1.19)
Prepregnancy BMI		
underweight	1.26(1.10–1.44)	1.26(1.10–1.44)
normal weight	1.00	1.00
overweight	0.94(0.85–1.03)	0.97(0.88–1.07)
obesity class I	0.79 (0.67–0.93)	0.82(0.70–0.97)
obesity class II	0.75 (0.54–1.02)	0.79(0.57–1.09)
obesity class III	0.44(0.21–0.93)	0.48(0.23–1.03)

^1^ Adjusted for maternal age, education, occupation, parity, plurality, civil status, smoking status during pregnancy, sex of the child, time period, and BMI

Women with a prepregnant BMI less than 18.5 kg/m^2^ had a greater risk of developing HG than women with a normal weight did in both the crude and adjusted analyses (adjusted OR (aOR) = 1.26; 95% CI: 1.10 − 1.44). Women with obesity class I (BMI = 30.0–34.9 kg/m^2^) and obesity class III (BMI > 40 kg/m^2^) were less likely to have hyperemesis than the reference group (cOR = 0.79; 95% CI: 0.67 − 0.93 and cOR = 0.44; 95% CI: 0.21 − 0.93, respectively) in crude analysis. The associations between overweight, obesity class II and HG did not reach statistical significance. The revealed associations only slightly changed after adjustment for the other studied factors, although the association between HG and obesity III was reduced to a nonsignificant level.

## Discussion

Our results fill an important gap concerning the prevalence of HG in the studied Russian population. The burden of illness caused by HG provides important information for healthcare planners and workers in planning medical care activities, developing preventive measures and assessing the effectiveness of these activities, which may decrease health care costs and time spent by women out of work.

The aim of this study was to investigate the prevalence of HG in the Murmansk and Arkhangelsk regions. We found that the prevalence of HG was 2.4%, which is within the range of 0.3% and 3.9% reported in other countries [18, 19]. The prevalence of HG in Northwest Russia is greater than that in neighboring Nordic countries but lower than that in Malaysia in Southeast Asia [22]. The differences in the prevalence of HG could be at least partially explained by access to healthcare services and inequalities in its utilization, different systems of data collection and diversity in diagnostic criteria [20, 21, 23, 29]. The Russian Society of Obstetricians and Gynecologists recommends the International Classification of Disease-10 (ICD-10) for classification and diagnoses of HG [30].

According to the ICD-10 classification O21.0 is mild HG and O21.1 is HG with metabolic disturbance, that starts before the end of the 22nd week of gestation and includes disturbance as: carbohydrate depletion, dehydration, electrolyte imbalance [30]. The Windsor definition for HG requires the following criteria: symptom starts before 16 weeks of gestation, are characterized by severe nausea and/or vomiting, inability to eat and/or drink normally and severely limits daily living activities [4]. The Windsor definition can be used for future studies to clarify on eligibility of patients in studies and as a guideline for clinicians diagnosing HG beyond the basic ICD-10 code.

The genetic origin of HG was supported by numerous studies [9, 10, 31]. GDF15, a stress response hormone that act on the brainstem and cause emesis, is highly produced by the placenta and growth rapidly in maternal blood during pregnancy [10]. Contrariwise, insufficient GDF15 serum level seems to be associated with miscarriage [32], so GDF15 may provide some benefits to the fetus, and might influence on the pregnant woman to avoid certain food that cause nausea and decreased appetite [33]. According to Fejzo et al., a family history of HG is a risk factor: 28% of diagnosed women had mothers with HG, and 19% of sisters had HG symptoms [34]. 

Variations in HG over time were found, which is in agreement with other studies [11, 12, 15, 21, 23]. In a Finnish study, the annual increase in HG 1 case/10 000 deliveries was observed from 2005 to 2017, but the reasons behind this increase are not known [15]. In a study from England, hospital admissions and antiemetic prescribing in HG cases increased continuously from 1998 to 2013 and were explained by better diagnostic procedures and increasing confidence in doctors’ prescribing [21]. In a Canadian study, decreasing antenatal admission rates for HG over time were explained by possible increased medication use for nausea and vomiting and prevention of HG development and a decreased number of deliveries to younger women and a greater tendency toward outpatient care in recent years [11]. We suppose, that the variations in HG over time in our study could be partly explained by the diversity in diagnostic criteria. The 2020 Russian clinical guidelines in gynecology include the HG classification, diagnostic criteria (persistent nausea and vomiting not associated with other causes, ketonuria associated with fasting, loss of body weight of at least 5%, electrolyte disturbances, hyperthyroidism, liver dysfunction), and methods of treatment [35]. Previously, only the Order of the Ministry of Health of the Russian Federation, November 7, 2012, No. 593n “Approval of the standard of specialized medical care for vomiting of pregnant women”, which was revised in 2013 without strict diagnostic criteria, existed [36]. Also, we can suppose higher prevalence of outpatient care with increased proportion of women registered in early pregnancy and increased medication use to prevent HG development after 2014.

In our study, the highest prevalence of HG was observed in women younger than 25 years. According to the adjusted analysis, these women were more likely to develop HG than older women were. The risk of HG decreased with increasing age, although the results did not reach statistical significance. These results are in line with previous literature: maternal age groups less than 20 and 20–24 years were risk factors for HG appearance, and in the maternal age group older than 30 years, the risk of HG decreased [11, 12, 15]. 

In our study, women and those with vocational or higher education demonstrated a high prevalence of HG in the crude analysis, but after adjustment, this association was not statistically significant. In a study concerning Norwegian-born women, there was no difference in the risk of HG occurrence by educational attainment [12]. In our study, occupational status was associated with a greater risk of HG even after adjustment. In a Finnish study, an analysis of cases of HG-affected pregnancies compared with non-HG pregnancies revealed that the risk of HG was lowest in non-employed women [15]. In some studies, the socioeconomic status of women was categorized in accordance with the average price of housing [14], demonstrating a greater risk of HG in women with lower socioeconomic status. In Russia, it is quite difficult to assess socioeconomic status on the basis of educational level and employment status, as some women may have a low level of education and not work outside the home but have high socioeconomic status and vice versa.

Married women presented a greater prevalence of HG, whereas unmarried and cohabiting women presented a lower risk for HG in the multivariable analysis. This finding is consistent with the results of a study from Norway [12] and contradicts the findings of an epidemiological study from California [20] and Germany [13]. In a Finnish study, marital status was not significantly associated with HG pregnancies in multivariable analysis [15]. 

Primiparity was associated with a greater risk of HG even after adjustment for potential confounders. A number of studies from different countries have noted an increased risk of HG among primiparous women [11, 14, 15]. Presumably, HG may influence women to avoid more than one pregnancy, due to their previous experiences of sever HG. Additionally, it can be assumed that in subsequent pregnancies, a woman may begin treatment earlier in the case of known symptoms occurring to stop the development of a serious disease.

The female sex of the child was associated with a greater likelihood of experiencing HG after adjustment for all the other studied factors. This finding is in accordance with the results of other studies [12, 14, 15]. GDF15 is considered to be the most likely cause of HG [31] and previous research confirm higher levels of GDF 15 in case of female fetus. Previously, maternal hormonal levels (estrogen and human chorionic gonadotropin (hCG)), associated with female offspring were considered to play a role in HG occurrence [2, 37, 38]. 

In our study, plurality was not significantly associated with HG, possibly due to the small number of multiple pregnancies in the study population. However, pregnancies with multiple fetuses have been consistently found to be associated with an increased risk of HG [11, 12, 15].

Nonsmoking women were more likely to have HG after adjustment for all factors in our study, which is in line with the literature [11, 17, 18]. That could be partly explained by maternal sensitivity to GDF15, when high levels of prepregnancy GDF15 are associated with reduced risk of HG [10].

The studies report that prepregnancy cigarette smoking was associated with elevated levels of GDF15 [39] and prepregnancy smoking reduces the risk of HG [18].

Underweight women presented a greater prevalence of HG, and women with class I obesity were less likely to have HG in our study. A number of authors reported that women with a low prepregnancy BMI had an increased risk of developing HG and that obesity was a protective factor [38, 40]. In other studies, the opposite was observed, and both low and high BMIs have been associated with a greater risk of HG [15, 17]. Another study demonstrated the correlation of GDF15 with BMI, while low GDF15 was associated with a lower incidence of nausea and pregnancy with a male fetus [37]. The level of GDF15 in blood increases during the pregnancy, and the increase is greater in normal weight woman than in obese; that is needed to ensure lowered glucose level and increased insulin secretory function in obese woman to maintain normoglycemia during pregnancy-induced insulin resistance [37]. That could be one of the possible explanation of reduction in odds of HG in association with increased BMI.

Thus, various explanations, associations, and risk factors for HG are found in the literature.

Following up on the prevalence of HG in the region in the future will be of interest for healthcare workers and planners to improve methods of treatment and decrease the burden of disease.

### Strengths and limitations

To our knowledge, this study is the first to investigate the prevalence and correlates of HG in Russia. The main strengths of this study include its large sample size and the use of registry-based data. The MCBR and the ACBR are reliable and validated sources of information [25, 26] that can be used to estimate the social and health status of pregnant women and data on the delivery and condition of babies in Russia. The coverage of births by the MCBR was 98.9%, and that by the ACBR was 99.6% of all births, resulting in minor selection bias. Information on sociodemographic and lifestyle characteristics included in registries is thoroughly collected, allowing us to perform a unique investigation of HG prevalence by maternal sociodemographic characteristics.

Regarding the limitations of the study, some cases of HG may not have been diagnosed and were not included in the registry if those women did not seek medical assistance or if the symptoms had already resolved by the time of medical visit. Thus, to meet this limitation, women whose first antenatal visit occurred after 22 weeks of gestation were excluded. HG cases in terminated pregnancies were not included in the study, as the registries collected data only on all births after 22 weeks of gestation. The prevalence of HG may be slightly underestimated in our study, as HG is mainly treated in hospitals, but some cases could be treated in private healthcare facilities that are not included in the registries. The information on maternal smoking was based on self-reported data, which may result in underestimation of smoking rates and could generate informational bias. All births were included in the registry, and more than one registered pregnancy per women would slightly cause overestimation of HG prevalence since in the case of HG occurrence in both pregnancies that will be counted twice, although this is the single patient. There were missing data in our registries, especially regarding smoking, first antenatal visit and maternal weight, with a consequent risk of selection bias and underestimation of the HG rate. The maternal prepregnancy BMI was estimated on the basis of BMI at the first antenatal visit with different timing of such visit, that could affect categorization of BMI, with a consequent risk of selection bias and underestimation of the HG rate. We studied the prevalence of HG on the basis of birth registries with the coverage of 98.9% births by the MCBR and 99.6% births by the ACBR. Meaning that a few pregnancies were missing and thus the severity of any potential HG were not captured in the registries. Etiological depth of HG is limited due to absence of clinical severity measures or GDF15 biomarkers in the study.

## Conclusions

The prevalence of HG in Russia from 2006 to 2018 was comparable with that reported in other countries, despite different diagnostic approaches and absence of international diagnostic criteria before 2021. The changes in the prevalence of HG over time in Russia cannot be explained only by maternal sociodemographic shifts examined in this study, plurality, parity, sex of the child, maternal smoking status and BMI. Our results confirm that the etiology of HG is multifactorial. The defined sociodemographic characteristics can be used for identification of risk groups and prevention of HG occurrence.

## Data Availability

The datasets analyzed during the current study are available from the Medical Analytical and Informational Centre of Arkhangelsk through the corresponding author on reasonable request.
